# Binding of Gamma-Glutamyl Transferase to TLR4 Signalling Allows Tissue Factor Activation in Monocytes

**DOI:** 10.3390/ijms232012207

**Published:** 2022-10-13

**Authors:** Chiara Sanguinetti, Valentina Scalise, Tommaso Neri, Alessandro Celi, Vanessa Susini, Maria Franzini, Roberto Pedrinelli

**Affiliations:** 1 Department of Translational Research and New Technologies in Medicine and Surgery, University of Pisa, 56126 Pisa, Italy; 2 Department of Surgery, Medical, Molecular, and Critical Area Pathology, University of Pisa, 56126 Pisa, Italy; 3 Istituto Nazionale per le Ricerche Cardiovascolari (INRC), 40126 Bologna, Italy

**Keywords:** tissue factor, gamma-glutamyl transferase, toll-like receptor 4, monocytes, coagulation, cytokine, atherosclerosis

## Abstract

Gamma-glutamyl transferase (GGT) is involved in the progression of atherosclerosis, since its enzymatic activity promotes the generation of reactive oxygen species (ROS). Besides, GGT may act as a prothrombotic factor by inducing tissue factor (TF) expression, independently of its enzymatic activity. The aim of this study was to assess whether GGT-induced TF stimulation was a consequence of binding to toll-like receptor 4 (TLR4) expressed on monocytes, the precursors of macrophages and foam cells which colocalize with GGT activity within atherosclerotic plaques. Experiments were performed in human peripheral blood mononuclear cells (PBMCs), THP-1 cells (a monocytic cellular model), and HEK293 cells, which were genetically modified to study the activation of TLR4. TF procoagulant activity was assessed by a one-stage clotting time test, and TF protein expression was estimated by western blot. Human recombinant (hr) GGT protein increased TF procoagulant activity and protein expression in both PBMCs and THP-1 cells. The GGT-induced TF stimulation was prevented by cellular pretreatment with TLR4/NF-κB inhibitors (LPS-Rs, CLI-095, and BAY-11-7082), and HEK293 cells lacking TLR4 confirmed that TLR4 is essential for GGT-induced activation of NF-κB. In conclusion, hrGGT induced TF expression in monocytes through a cytokine-like mechanism that involved the activation of TLR4/NF-κB signaling.

## 1. Introduction

Serum γ-glutamyl transferase (GGT) is a single-pass type II membrane glycoprotein localized on the apical surfaces of various cells [1]. GGT is a well-established biomarker for hepatobiliary disease and alcohol-related liver diseases. However, elevated serum GGT levels have been also associated to increased risk of several disorders [2], including cardiovascular diseases (CVDs) [3,4]. Indeed, evidence from large epidemiological studies strongly suggests the existence of an association between elevated serum GGT activity and coronary heart disease (CHD), arterial hypertension, congestive heart failure, cardiac arrhythmias, and CVD-related mortality [3,4,5,6]. However, the underlying mechanisms of this association are still unknown.

The presence of GGT activity in atherosclerotic plaques and its correlation with histological indexes of plaque instability may suggest a role of GGT in the pathophysiology of CVD related to atherosclerosis. Indeed, GGT is likely involved in the progression of atherosclerosis, since its enzymatic activity promotes the generation of reactive oxygen species (ROS) within the plaque, thus contributing to oxidative reactions such as peroxidation of cellular membranes and of low-density lipoproteins [7,8,9]. Currently, this mechanism is the most largely accepted for direct participation of GGT in the pathophysiology of atherosclerosis [7,10,11,12].

Recently, it has been proposed that GGT may act as a prothrombotic factor, independently of its enzymatic activity [13]. This study was conducted using a human recombinant GGT protein (hrGGT) synthesized by wheat germ eukaryotic translational apparatuses in which the lack of post-translational glycosylation forbids the generation of enzymatically active GGT [14,15]. We have previously reported that hrGGT induces the gene expression of tissue factor (TF) and TF-related procoagulant activity in peripheral blood mononuclear cells (PBMCs) [13]. Among PBMCs, monocytes are the most probable source of TF procoagulant activity [16]. In atherosclerotic plaques, monocytes-derived cells express both TF [17] and GGT [18], so the latter could contribute to the progression of atheroma both through ROS production and TF expression.

Toll-like receptor 4 (TLR4) is a well-known receptor of the pattern recognition receptor family which is expressed in myeloid cells. TLR4 activation triggers a signal cascade that leads to the nuclear translocation of the transcription factor nuclear factor-κB (NF-κB), which in turn induces the expression of pro-inflammatory cytokines [19], as well as that of TF [20]. TLR4 has several ligands, both pathogen (i.e., lipopolysaccharide (LPS)) and endogenous-derived [21,22,23]. Interestingly, GGT has been identified as a TLR4 ligand in studies conducted on osteoclast cells [24] which share their ontogenesis with monocytes [25]. This study aimed at confirming the involvement of monocytes in GGT-induced TF procoagulant activity, as previously hypothesized [13], and to investigate the involvement of TLR4 signaling in hrGGT induction of TF-related procoagulant activity.

## 2. Results

### 2.1. hrGGT Stimulates TF Activity in PBMCs and THP-1 Cell Lines

**Figure 1:**
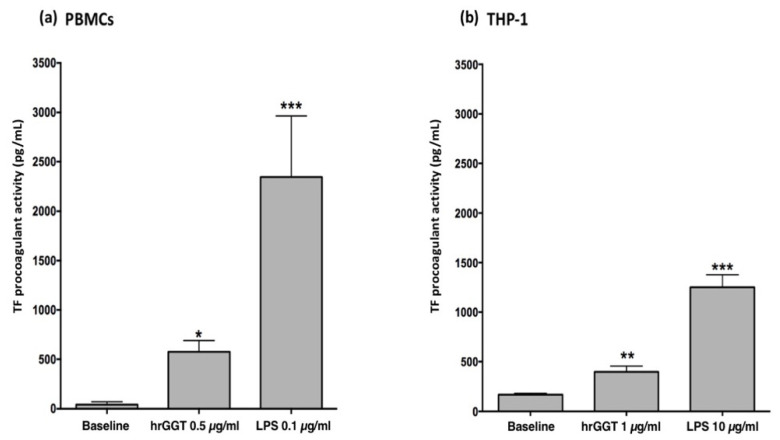
(**a**) TF procoagulant activities measured in PBMCs stimulated by hrGGT (0.5 µg/mL) and LPS (0.1 µg/mL). (**b**) TF activity measured in THP-1 cells stimulated by hrGGT (1 µg/mL) and LPS (10 µg/mL). Data are presented as means ± SEM (*n* = 10). * *p* < 0.05 vs. the baseline; ** *p* < 0.01 vs. the baseline; *** *p* < 0.001 vs. the baseline.

First of all, it was verified if hrGGT, a molecule devoid of enzymatic activity, could stimulated TF expression in THP-1 cells, a cellular model widely used to study monocytes functions and responses. The results showed an increase of TF procoagulant activity in THP-1 as previously reported in PBMCs (Figure 1), although a higher concentration was required to induce a response from THP-1 (1 µg/mL hrGGT and 10 µg/mL LPS) in comparison with that from PBMCs (0.5 µg/mL hrGGT and 1 µg/mL LPS).

**Figure 2:**
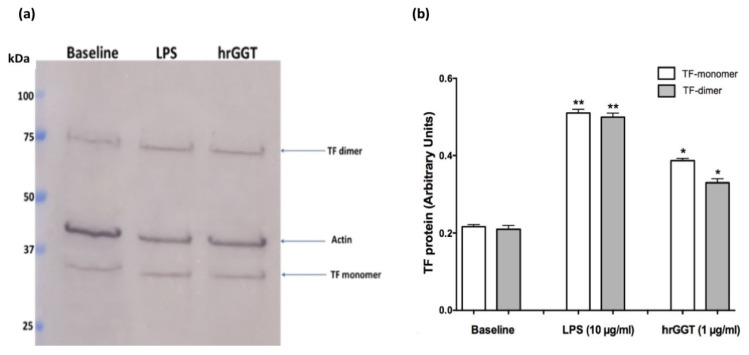
hrGGT induces TF protein expression in THP-1 cells. Panel (**a**) Representative western blot image of TF expression (monomers at ~35 kDa and dimers at ~75 kDa) in response to hrGGT (1 µg/mL). Panel (**b**) Semi-quantitative densitometric analysis (ImageJ software) of TF monomer and dimer expression. Data are reported in arbitrary units normalized to the corresponding β-Actin signals. LPS (10 µg/mL) was used as a positive control. Data are presented as mean ± SEM (*n* = 3) for Western blot experiments. * *p* < 0.05 vs. the baseline; ** *p* < 0.01 vs. the baseline.

Western blot analysis was performed to ascertain if the increased TF procoagulant activity in THP-1 was consequent to an increase of TF protein expression, as already shown for PBMCs [13]. Cell treatment with LPS (10 µg/mL) represented the positive control for TF expression (Figure 2).

### 2.2. LPS Contamination Does Not Explain GGT-Induced TF Activity

**Figure 3:**
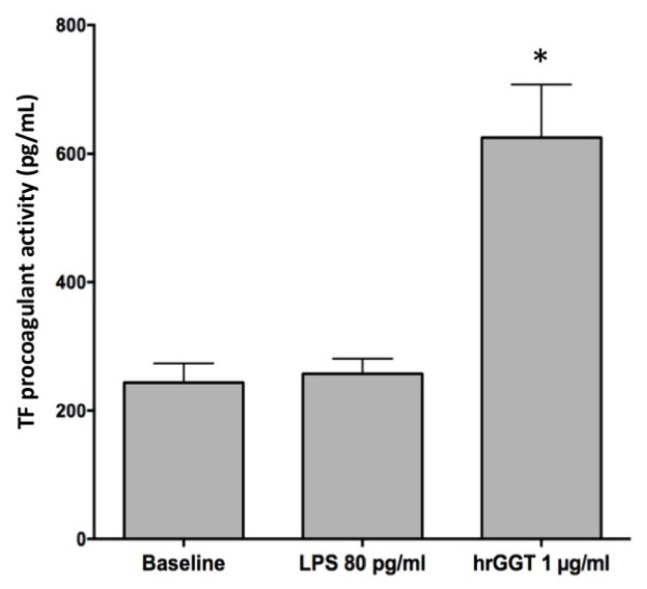
TF procoagulant activities measured in THP-1 cells stimulated by hrGGT (1 µg/mL) and LPS (80 pg/mL). Data are presented as means ± SEM (*n* = 3). * *p* < 0.05 vs. LPS. A multiple comparison was performed by the Friedman test.

The LAL assay revealed the presence of LPS (80 pg/mL) in a 1 µg/mL hrGGT incubation mixture. To verify the possible interference of LPS on TF expression, we treated THP-1 cells lines with that same contaminant LPS concentration (80 pg/mL LPS), but as reported in Figure 3, LPS amount did not affect the baseline TF activity, which was quite different from hrGGT stimulation used as a comparison (Figure 3).

### 2.3. Specificity of TF Procoagulant Activity

**Figure 4:**
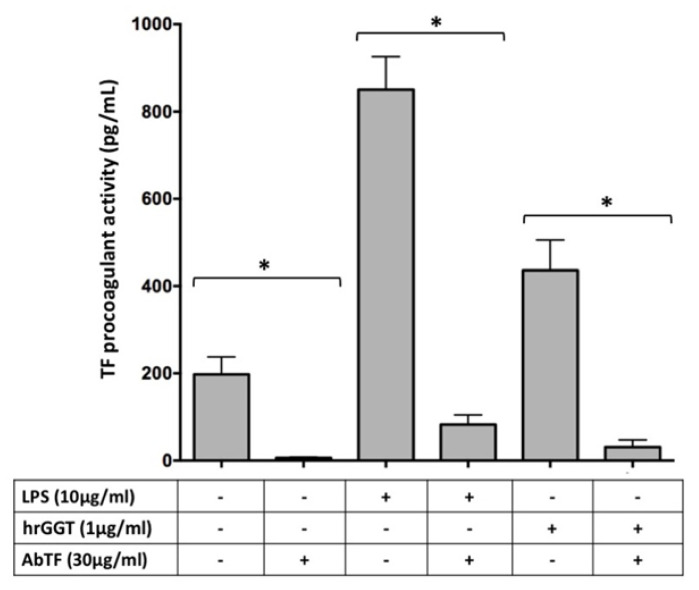
TF procoagulant activities measured in THP-1 cells stimulated by hrGGT (1 µg/mL) and LPS (10 µg/mL) in the presence or absence of an anti-TF antibody (30 µg/mL). Data are presented as mean ± SEM (*n* = 3). * *p* < 0.05. The Wilcoxon test was used.

To confirm that hrGGT-induced procoagulant activity was TF-dependent, THP-1 cells were pre-incubated with a specific human anti-TF antibody (30 µg/µL), which abolished hrGGT-induced TF procoagulant activity (Figure 4), as previously observed in PBMCs [13].

**Figure 5:**
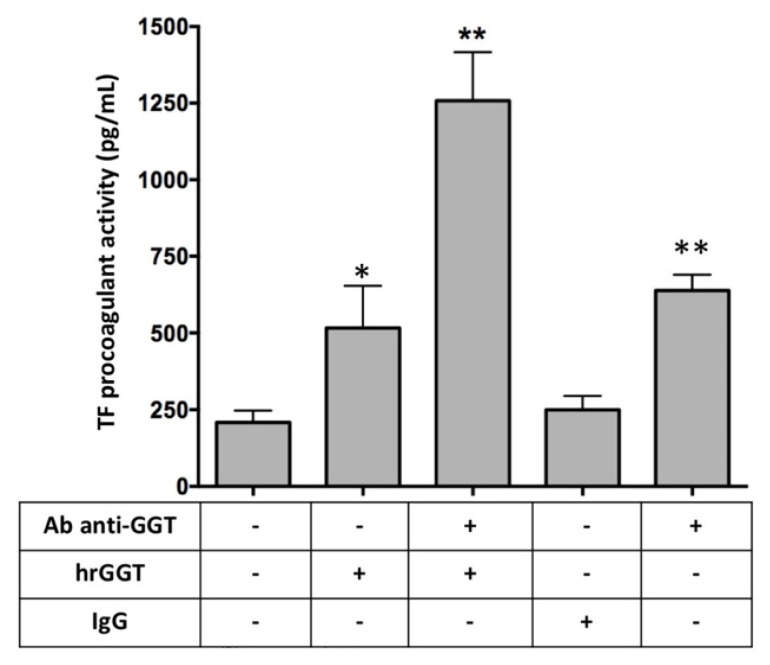
TF procoagulant activities measured in THP-1 cells stimulated with 1 µg/mL hrGGT, a 5 µg/mL anti-GGT antibody alone, and with hrGGT. Human IgG (5 µg/mL) was used as a control. Data are presented as mean ± SEM (*n* = 3). * *p* < 0.05 vs. the basal condition; ** *p* < 0.01 vs. the basal condition. A multiple comparison was performed by the Friedman test.

To test the specificity of hrGGT in inducing TF procoagulant activity, THP-1 cells were pre-treated with an anti-GGT antibody. Unexpectedly, the subsequent addition of hrGGT induced a greater increase of TF procoagulant activity compared to the treatment with only hrGGT (Figure 5).

### 2.4. Cell Vitality: MTT Assay on THP-1 Cell Lines

**Figure 6:**
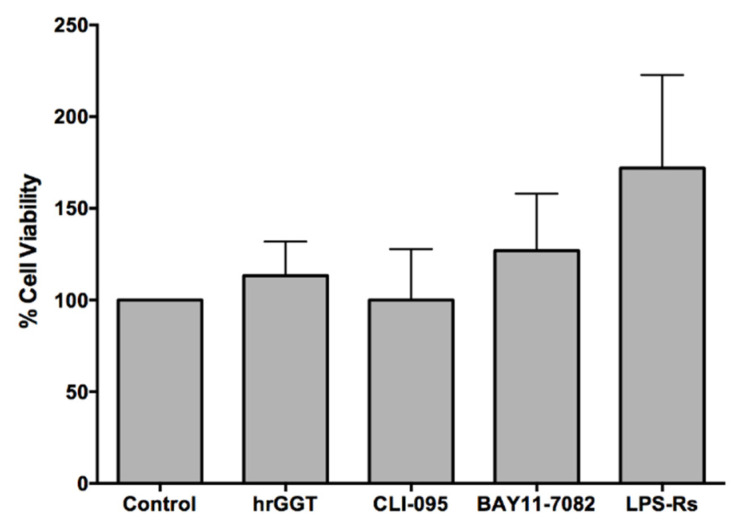
MTT assay in THP-1 cells after 4 h treatment with hrGGT (1 µg/mL) and several TLR4 signaling inhibitors (10 µg/mL LPS-Rs, 3 × 10^−6^ M CLI-095, and 10^−5^ M BAY-11-7082). Cell viability was reported as the absorbance ratio (expressed as a percentage) of treated cells to untreated cells. Data are presented as mean ± SEM (*n* = 3).

A viable MTT assay was performed to verify if TLR4 inhibitors were cytotoxic for THP-1 cells at the chosen work concentrations (Figure 6). The results showed no signs of adverse effects after treatment with either hrGGT (1 µg/mL) or TLR4 signaling inhibitors (10 µg/mL LPS-Rs, 3 × 10^−6^ M CLI-095, and 10^−5^ M BAY-11-7082).

### 2.5. Inhibition of the TLR4/NF-κB Pathway Downregulates LPS- and hrGGT-Induced TF Procoagulant Activity

**Figure 7:**
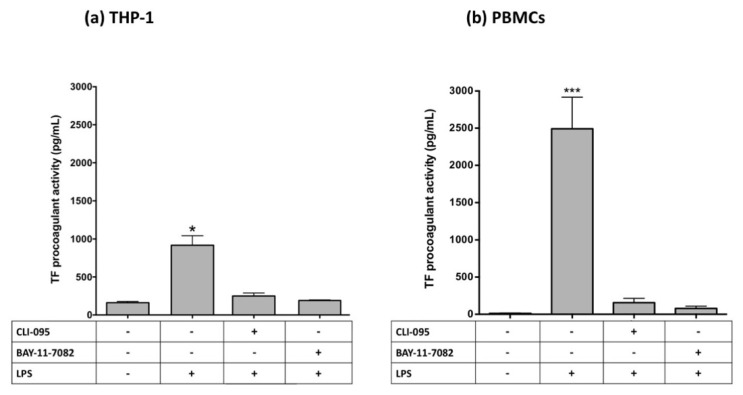
TF procoagulant activities measured in THP-1 (**a**) and PBMCs (**b**). Both cell lines were pre-treated with specific inhibitors (3 × 10^−6^ M CLI-095 and 10^−5^ M BAY-11-7082) for 30 min and subsequently stimulated with LPS (10 µg/mL for 4 h for THP-1 and 0.1 µg/mL for 18 h for PBMCs). Data are presented as mean ± SEM (*n* = 8). * *p* < 0.05 vs. the basal condition; *** *p* < 0.001 vs. the basal condition.

**Figure 8:**
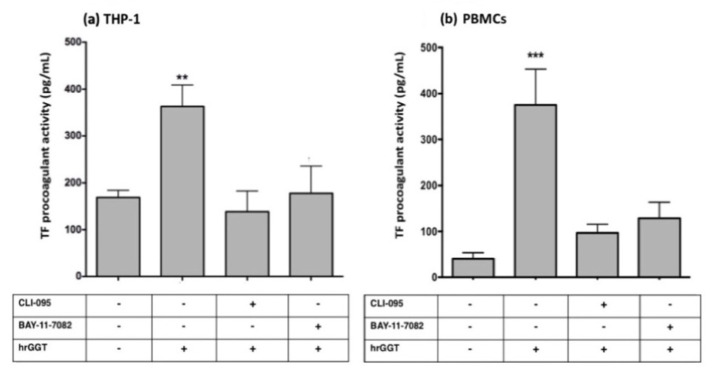
TF procoagulant activities measured in THP-1 (**a**) and PBMCs (**b**). Both cell lines were pre-treated with specific inhibitors (3 × 10^−6^ M CLI-095 and 10^−5^ M BAY-11-7082) for 30 min and subsequently stimulated with hrGGT (1 µg/mL for 4 h for THP-1 and 0.5 µg/mL for 18 h for PBMCs). Data are presented as mean ± SEM (*n* = 8). ** *p* < 0.01 vs. the basal condition; *** *p* < 0.001 vs. the basal condition.

**Figure 9:**
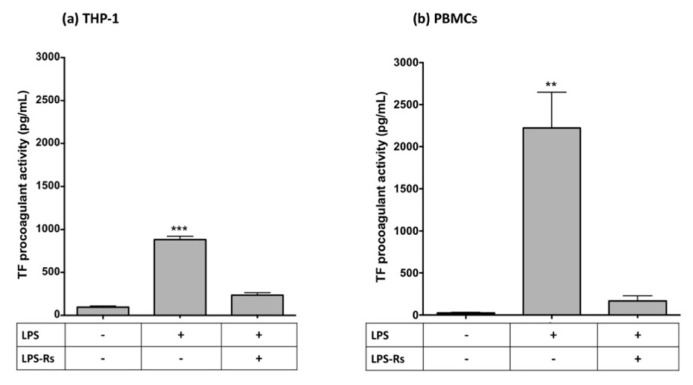
TF procoagulant activities measured in THP-1 (**a**) and PBMCs (**b**). Both cell lines were pre-treated with a specific TLR4 antagonist (i.e., 1 µg/mL LPS-Rs) for 30 min and subsequently stimulated with LPS (10 µg/mL for 4 h for THP-1 and 0.1 µg/mL for 18 h for PBMCs). Data are presented as mean ± SEM (*n* = 4). *** *p* < 0.001 vs. the basal condition; ** *p* < 0.01 vs. the basal condition.

**Figure 10:**
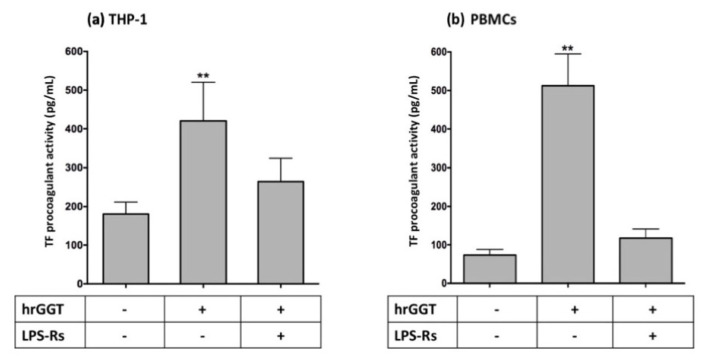
TF procoagulant activities measured in THP-1 (**a**) and PBMCs (**b**). Both cell lines were pre-treated with a specific TLR4 antagonist (i.e., 1 µg/mL LPS-Rs) for 30 min and subsequently stimulated with hrGGT (1 µg/mL for 4 h for THP-1 and 0.5 µg/mL for 18 h for PBMCs). Data are presented as mean ± SEM (*n* = 4). ** *p* < 0.001 vs. the basal condition.

In both cell lines, CLI-095 (a TLR4 signaling inhibitor) and BAY11-7082 (a specific NF-κB inhibitor [26]) completely inhibited TF procoagulant activity induced by both LPS (Figure 7) and hrGGT (Figure 8). Furthermore, both cell lines were treated with LPS-RS, a TLR4 extracellular antagonist which abolished both LPS- (Figure 9) and hrGGT-mediated TF procoagulant activity (Figure 10).

### 2.6. Activation by hrGGT of NF-κB through TLR4 Signaling in HEK-Blue Cells

**Figure 11:**
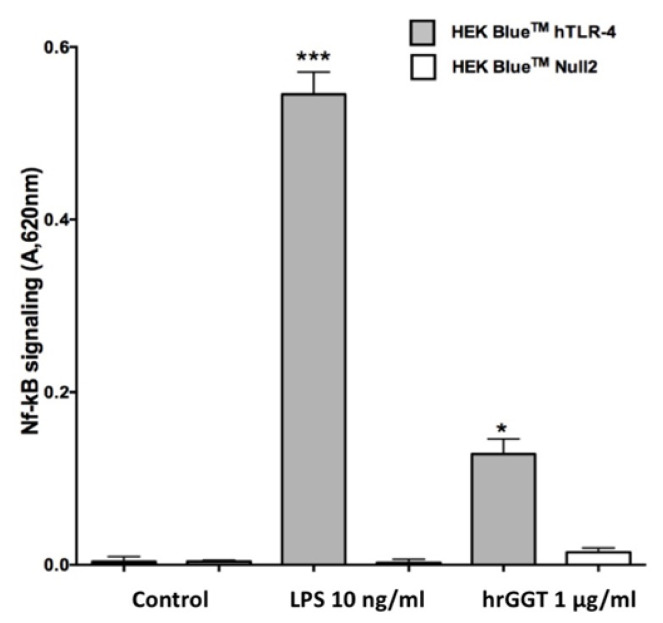
NF-κB quantification in HEK-Blue hTLR4 and HEK-Blue Null2 cells stimulated with 1 µg/mL hrGGT for 18 h at 37 °C and at an atmosphere of 5% CO_2_. hrGGT induced NF-κB in HEK-Blue hTLR4 cells, but not in HEK-Blue Null2 cells which lacked TLR4 receptor. LPS (10 ng/mL), the selective agonist of TLR4s, was used as a positive control. *** *p* < 0.001 vs. control; * *p* < 0.05 vs. control. Data are presented as mean ± SEM (*n* = 3). The relative expression level of SEAP (directly proportional to Nκ-kB activity) was determined spectrophotometrically at 620 nm.

The demonstration that hrGGT could induce NF-κB activation was obtained by treating HEK-Blue hTLR4/MD-2/CD14 cells with 1 µg/mL hrGGT. The same stimulation on HEK-Blue Null2 cells, which did not express TLR4 complex, confirmed the role of TLR4 signaling for GGT-induced NF-κB stimulation (Figure 11).

## 3. Discussion

The original findings obtained in this study allowed a better understanding of the hrGGT procoagulant role, previously shown in our previous study conducted in PBMCs [13].

PBMCs preparation typically contains 25–35% monocytes and 65–75% lymphocytes with a minimal proportion of neutrophils (>5%). Since it has been shown that the presence of lymphocytes and neutrophils did not influence the procoagulant activity of monocytes [16,27,28], all experiments were performed in PBMCs without the exclusion of lymphocytes. To confirm the procoagulant role of monocytes, the experiments performed on PBMCs [13] were repeated on THP-1 cells, the cellular line widely used as a monocytic model. The collected data confirmed that hrGGT induced TF expression in monocytic cells. Interestingly, hrGGT was devoid of enzymatic activity; thus, we confirmed that GGT has a cytokine-like function as previously suggested [24]. Furthermore, our results showed that hrGGT-stimulated TF expression was dependent on the TLR4/NF-κB signaling pathway.

### 3.1. GGT Upregulates Monocytic TF Expression

Similar to what was described in PBMCs [13], hrGGT stimulation of THP-1 cells increased both TF protein expression and procoagulant activity, with the latter being wholly ascribed to TF/FVIIa interaction since an anti-TF antibody abolished that response. Unfortunately, it was impossible to prove the specificity of hrGGT action with an anti-GGT antibody. Indeed, contrary to the expected, the pre-incubation of cells with an anti-GGT antibody amplified the TF procoagulant activity as compared to hrGGT per se. It is known that immunocomplexes can activate TLR4 [29]. Furthermore, monocytes express neonatal Fc receptor for IgG (FcRn) and Fc gamma receptor (FcγR) families whose activation by single IgG and, to a greater extent, immunocomplexes IgG activates TF gene transcription [30,31]. This appears to be in agreement with our results: treatment with an anti-GGT antibody alone induces TF expression which doubles after the addition of a specific antigen (Figure 5). Furthermore, it is known that the affinity of IgG to FcγR is influenced by specific antibody glycosylation patterns which change according to different antibodies production conditions [32].

To exclude any possible interference by LPS, the TLR4 natural ligand, its amount in reagents, solutions, and cell cultures were scrupulously checked with LAL assay at all stages of the experimental designs. A small quantity of LPS in hrGGT preparation was actually detected, but it was irrelevant in the used experimental conditions. Thus, the observed increases in TF expression and activity were dependent on hrGGT stimulus.

### 3.2. hrGGT Activates TLR4/NF-κB Signaling

In PBMCs, it was shown that an hrGGT-related TF expression enhancement was achieved through NF-κB activation [13]. Indeed, the incubation of PBMCs with an NF-κB inhibitor (BAY-10-772) prevented both hrGGT- and LPS-induced increases of TF procoagulant activity [13].

On this basis, the relationship between hrGGT extracellular stimulus and NF-κB activation was investigated in THP-1 cells. Since one of the best characterized triggers for NF-κB activation involves TLR4 activation [33,34], we investigated whether hrGGT influenced this pathway by evaluating the effects of BAY-10-772 and other two inhibitors of the TLR4 signaling pathway: LPS-RS which is a TLR4 antagonist [35] and CLI-095 which is a pharmacological probe that disrupts the interaction of endogenous adaptors to the TIR complex [36]. The obtained result confirmed the hypothesis that GGT protein could be an activating ligand for TLR4, since the different inhibitors of TLR4 signaling abrogated hrGGT-induced TF procoagulant activity in both PBMCs and THP-1 monocytes. In this latter regard, the reduced sensitivity of THP-1 cells to hrGGT stimulation as compared to PBMCs could be explained by the low expression of the TLR4 signaling complex in THP-1 monocyte-like cells [37].

Experiments on the HEK-Blue hTLR4 cells showed that hrGGT could activate NF-κB by engaging the functional TLR4/MD-2/CD14 complex expressed by these engineered cells although to a lesser extent than LPS, the selective agonist of the TLR4 complex. As a counterproof, both hrGGT and LPS did not activate NF-κB in TLR4-negative HEK-Blue Null2 cell lines despite a maintained responsiveness of the system to tumor necrosis factor alpha (TNFα), an NF-κB agonist acting through an independent signaling pathway [38].

## 4. Materials and Methods

### 4.1. Reagent and Materials

RPMI-1640, DMEM, blasticidin, penicillin, streptomycin, Ficoll-Hypaque, sodium citrate, LPS from *Escherichia coli* O55:B5 applied without repurification, hrGGT, BAY-11-7082 (BAY), TF, TNFα, and β-actin were purchased from Sigma-Aldrich, Milan, Italy. An hrGGT stock (Abnova, Taipei City, Taiwan) was prepared at 10 ng/mL in endotoxin-free water. A human anti-TF antibody (epitope specific for aa 1–25) and relipidated full-length recombinant human TF were obtained from BioMedica Diagnostics, Windsor, NS, Canada. Mini-PROTEAN TGX Gel, Precision Plus Protein All Blue, Trans-Blot Turbo transfer system, Opti-4CN substrate Kit, Goat Anti-Rabbit IgG H&L (HRP), and Goat Anti-Mouse IgG H&L (HRP) were obtained from Bio-Rad, Hercules, CA, USA. Ultrapure LPS-RS, CLI-095 (CLI), HEK293 human (h)TLR4-positive (HEK-Blue hTLR4), and negative (HEK-Blue Null2) cell lines were purchased from InvivoGen, Toulose, France. A GGT-1-purified MaxPab mouse polyclonal antibody (B01P) was purchased from Abnova, Taipei City, Taiwan. An LAL chromogenic endpoint assay was obtained from Hycult Biotech, Uden, The Netherlands.

### 4.2. Cell Culture

#### 4.2.1. PBMCs Preparations

Human PBMCs suspensions were obtained from buffy coats left over from blood bank draws and taken from healthy donors with the approval of the Ethics Committee of the Pisa University Hospital (protocol code: 558).

Buffy coats were kept at room temperature and used within 4 h from withdrawal. PBMCs were isolated by centrifugation on Histopaque-1077 at 400× *g* at a controlled temperature of 20 °C for 30 min. Cells collected from the interphase were washed twice in 0.38% sodium citrate and resuspended in RPMI-1640 medium supplemented with 1% penicillin-streptomycin. Drugs were kept in a stock solution and diluted in serum-free RPMI at appropriate concentrations immediately before use. Cell vitality was assessed by 3-(4,5-dimethylthiazol-2-yl)-2,5-diphenyltetrazolium bromide (MTT; >85% of viable cells), and the constancy of the cell number was verified at all experimental phases. The final PBMCs preparations typically contained 25–35% monocytes, negligible proportions of neutrophils (<5%), and 65–75% lymphocytes, and residual platelets were less than 1/mononuclear cell. The cellular content of PBMCs preparations was verified by the microscopic observation of May Grunwald Giemsa staining.

After isolation, cells were resuspended in polypropylene tubes (3 × 10^6^ cells/mL), pre-treated with the various pharmacological probes used in the study (see below) for 30 min prior to stimulation with 0.5 µg/mL hrGGT or 0.1 µg/mL LPS and then left in incubation at 37 °C for 18 h before testing TF procoagulant activity. The optimal stimuli concentrations and time of stimulation were chosen as previously described [13].

#### 4.2.2. THP-1 Cell Line as a Monocyte Response Model

Tsuchiya et al. established a human THP-1 cell line in 1980 from the peripheral blood of a 1-year-old human male with acute monocytic leukemia [39]. Early studies indicated that THP-1 cells resemble primary monocytes and macrophages in morphological and differentiation properties. Therefore, THP-1 cell lines have been widely used to study monocytes functions and responses.

A THP-1 cell line was purchased from the European Collection of Authenticated Cell Cultures (ECACC, 88081201) and was grown in RPMI 1640 culture medium supplemented with 10% fetal bovine serum, 2 mM L-glutamine, 1 mM sodium pyruvate, 100 U/mL penicillin, and 100 µg/mL streptomycin at 37 °C and at an atmosphere of 5% CO_2_. Cells were sub-cultured twice a week and were maintained in a logarithmic growth phase at a concentration of 3–5 × 10^5^ cells/mL.

For the procoagulant activity test, 4 × 10^5^ cells were incubated at 37 °C for 4 h with 1 µg/mL hrGGT or 10 µg/mL LPS as a final concentration in the medium. It should be noted that THP-1 required an hrGGT and LPS concentration for cells stimulation twice as high as that used in PBMCs [13]. This different behavior agrees with the literature data showing a lower sensitivity of THP-1 compared to the sensitivity of PBMCs in response to LPS. The longer incubation time of PBMCs compared to that of THP1 cells was due to the delayed delivery of buffy coats from the blood bank, which did not allow completing the experimental procedures in the same day. In preliminary experiments, PBMCs responses to hrGGT tested after 4 and 18 h incubation did not differ. Thus, the cells treated were then subjected to the evaluation of TF procoagulant activity using a STart Max semi-automated Coagulation analyzer.

#### 4.2.3. HEK-Blue Cells

HEK-Blue hTLR4 cells are HEK293 (Human Hepatic Embryonic Kidney (HEK)-293) cells specifically designed by InvivoGen for studying the stimulation of human TLR4 by monitoring the activation of NF-κB. HEK-Blue hTLR4 cells are stably transfected with two reporter constructs for the expression of TLR4/MD-2/CD14 co-receptor genes and that of an inducible reporter gene (i.e., secreted embryonic alkaline phosphatase SEAP). Only after TLR4 stimulation, NF-κB is activated, and then, the secretion of SEAP is promoted. The principle of SEAP determination is detailed within the cell line datasheet available at the link: https://www.invivogen.com/hek-blue-hTLR4 (accessed on 16 September 2022).

A HEK-Blue hTLR4 cell line was used to test hrGGT as a TLR4 agonist, while HEK-Blue Null2 cells were used as control cells as they lacked TLR4 receptor. Cell lines were purchased from InvivoGen and were cultured at 37 °C in Dulbecco’s minimal essential media (DMEM) containing 1% glutamine, 10% heat-inactivated fetal bovine serum (FBS), 1% penicillin/streptomycin, and 1% normocin.

### 4.3. TF Procoagulant Activity

TF procoagulant activity was assessed in PBMCs (1 × 10^6^ cells/mL) and THP-1 (4 × 10^5^ cells/mL) by a one-stage clotting time test using a STart Max semi-automated Coagulation analyzer (Diagnostica Stago S.A.S., Milano, Italy), as previously described [40]. Briefly, an equal volume of pooled normal human plasma was added to PBMCs or THP-1 suspensions (100 µL), and then, 25 mM CaCl_2_ was used to start thrombin generation; measurements were performed at 37 °C. For each experimental session, calibration curves were created using recombinant human relipidated TF as a standard. Clotting times (mean ± SD, *n* = 20 for each TF concentration) by increasing TF concentrations (0.001 pg/mL, 688 ± 84 s; 0.01 pg/mL, 386 ± 56 s; 0.1 pg/mL, 192 ± 29 s; 1 pg/mL, 84 ± 6 s; 10 pg/mL, 40 ± 3 s; 100 pg/mL, 19 ± 2 s). The inter-assay variation coefficient ranged from 7% to 15% (mean: 11%; 95% confidence interval: 7.5–14.7%). “Baseline” values refer to quiescent, non-activated, untreated cells with clotting times above 365 s and 162 s for PBMCs and THP-1, respectively. All experimental points (*n* = 20 for each TF concentration) were run in triplicate and averaged.

### 4.4. Western Blot Analysis

For TF analysis by western blot, THP-1 cells were treated with LPS or hrGGT as describe above. After 4 h of incubation, cells were pelleted and resuspended in Dulbecco’s phosphate-buffered saline (PBS, 25 µL) and lysed on ice for 30 min. The protein concentration of samples was determined using a Bradford assay [41]. Cell lysates were resolved in sodium dodecyl sulphate polyacrilamide gel electrophoresis (SDS-PAGE). For each sample, 50 μg of proteins were loaded on a 4–20% gradient Mini-PROTEAN TGX Gel (Bio-Rad), and Precision Plus Protein All Blue (Bio-Rad) was used as a standard. Separated proteins were transferred onto a polyvinylidene difluoride (PVDF) membrane using a transfer apparatus (Trans-Blot Turbo, Bio-Rad). The membrane was blocked with 5% skim milk and incubated first with an anti-TF primary antibody and then with an anti-actin primary antibody. After being washed three times, membranes were incubated with secondary antibodies: goat anti-mouse IgG H&L (HRP) and goat anti-rabbit IgG H&L (HRP) (1:1000). Protein detection was performed using a Bio-Rad Opti-4CN substrate Kit. Densitometry was performed using the open-source software ImageJ (version 1.51) [42]. The abundance of the TF protein was normalized to the total amount of the housekeeping protein (β-Actin) in each lane, and relative expressions were calculated in comparison to that of the control (baseline condition).

### 4.5. Evaluation of Endotoxin Contamination by a Limulus Amoebocyte Lysate (LAL) Assay

Since LPS contamination during experimental procedures is frequent and leads to erroneous result interpretation [43], all reagents and solutions used for cell isolation and cultures were prepared with endotoxin-free water and glassware was rendered endotoxin-free by exposure to high temperature. Moreover, GGT preparation and reagents and solutions used for in vitro cell cultures were preliminarily tested on a routine basis with a sensitive chromogenic LAL assay [44]. Briefly, all the reagents and cells used in experimental design were mixed with the LAL reagent (volume ratio: 1:1) and incubated at 37 °C for 15 min. The reaction was terminated by adding a blocking solution, and absorbance was measured at 405 nm using a microplate reader.

### 4.6. TLR4/NF-κB Pathway in hrGGT-Induced TF-Procoagulant Activity

**Figure 12:**
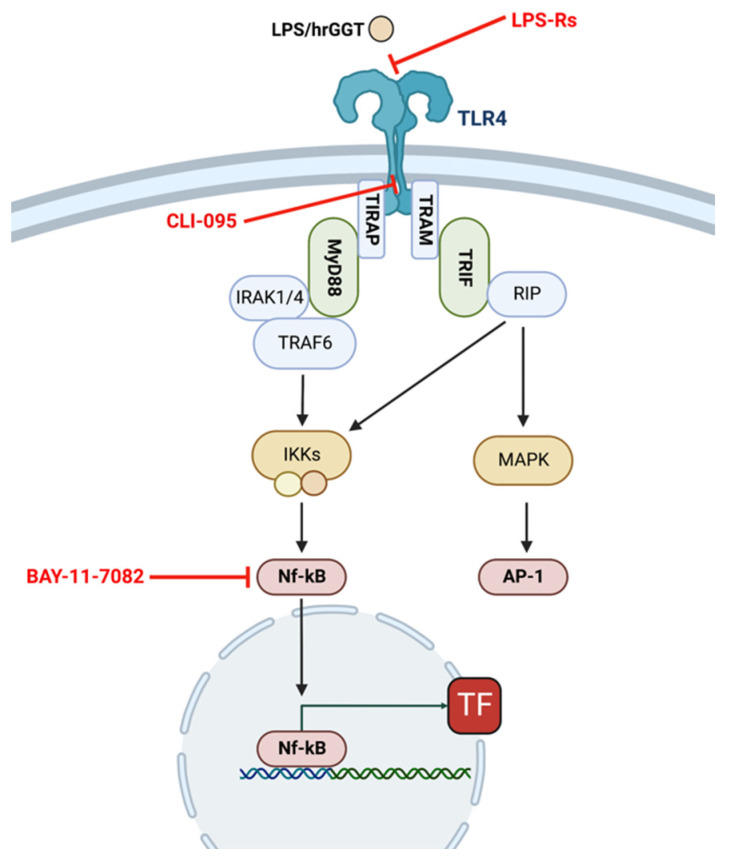
The NF-κB activation pathway via TLR4 and the mode of action of pharmacological probes formulated to block TLR4 receptor in different signaling steps. TLR4 activation by ligands (LPS or hrGGT) triggers the TLR4 signaling cascade that activates NF-κB, allowing it to translocate to the nucleus and activate TF gene transcription. Inhibitors are shown in red: LPS-Rs is an extracellular TLR4 antagonist, CLI-095 is an intracellular TLR4 signaling inhibitor, and BAY-11-7082 is a specific NF-κB inhibitor. The figure was created through the website “biorender.com”.

To investigate if the TLR4 pathway is involved in inducing TF expression after GGT stimulation, PBMCs and THP-1 were pre-treated 30 min with several pharmacological probes formulated to block TLR4 receptor in different signaling steps (Figure 12): 10 µg/mL LPS-Rs which is an extracellular TLR4 antagonist, 3 × 10^−6^ M CLI-095 which is an intracellular TLR4 signaling inhibitor, and 10^−5^ M BAY-11-7082 which is a specific NF-κB inhibitor. The used concentrations were chosen according to the manufacturer’s instruction.

### 4.7. Cell Vitality: Dimethyl Thiazolyl Diphenyl Tetrazolium (MTT) Assay

The MTT assay is widely used to measure cellular metabolic activity as an indicator of cell viability, proliferation, and cytotoxicity. The test was applied to evaluate the safety of hrGGT and TLR4 inhibitors on THP-1 cell lines or PBMCs. For this purpose, the cells were exposed for the same time (4 h and 18 h for THP-1 and PBMCs, respectively) and at the same concentration of stimuli and TLR4 inhibitors as previously described. After the incubation period, the cell solution was divided with 100 µL placed into each well of a 96 well-plate, and each well was incubated with 11 µL MTT for 4 h. Then, the cells were incubated with 111 μL of dimethyl sulfoxide (DMSO) for 10 min to dissolve formazan crystals. The absorbance of the MTT formazan was determined at 595 nm using a multi-well spectrophotometer. Viability was defined as the ratio of the absorbances of treated cells to untreated cells (expressed as a percentage).

### 4.8. NF-κB Reporter Assay

HEK-Blue hTLR4 cells and HEK-Blue Null cells were harvested for the experiment, when a 70% confluence was reached. The cells were then plated at a density of 2.5 × 10^4^ viable cells per well in 96-well plates in a 200 µL final volume and subjected to stimuli: hrGGT was added at the concentration of 1 µg/mL and LPS (100 ng/mL) was used as a positive control in HEK-Blue hTLR4 cells, while TNFα (100 ng/mL) was used as a positive control in HEK-Blue Null2 cells.

After cell stimulation, NF-κB activation was determined using a QUANTI-Blue detection reagent, a chromogenic substrate for SEAP, which allowed measuring spectrophotometrically (optical density at 620 nm) the SEAP activity accumulated in the culture medium after 18 h of incubation with stimuli.

### 4.9. Statistic Analysis

The statistical significances of between and among-groups differences were tested by the Kruskal−Wallis test unless otherwise indicated. Data were analyzed with GraphPad Prism 6 and expressed as mean ± SEM. *p* < 0.05 was considered as statistically significant.

## 5. Conclusions

In conclusion, hrGGT induced an increase of TF procoagulant activity and protein expression in both PBMCs and THP-1 monocytes and the activation of the TLR4/NF-κB signaling pathway, which represented the biological transducer of this phenomenon. The upregulation of TF in response to hrGGT, an enzymatic inactive protein, highlighted a new role for the GGT protein in the progression of cardiovascular diseases, independently of its enzymatic activity. In atherosclerotic disease, where the plaque environment includes GGT and TF-expressing monocytes, GGT could promote the disease progression not only by generating ROS derived from its enzymatic activity, but also by a cytokine-like mechanism that induces TF expression. Further studies will be needed to clarify the different contributions of the enzymatic and cytokine function of GGT in atherosclerotic disease. However, TF upregulation in response to hrGGT suggests a key role of the protein in the coagulation process and helps to better understand the still unclear mechanism correlating elevated serum GGT levels and cardiovascular diseases related to atherosclerosis [3,45].

## Data Availability

The datasets are available from the corresponding author on reasonable request.
